# Chloroquine augments TRAIL-induced apoptosis and induces G2/M phase arrest in human pancreatic cancer cells

**DOI:** 10.1371/journal.pone.0193990

**Published:** 2018-03-07

**Authors:** Hiroyuki Monma, Yuichi Iida, Tamami Moritani, Tamio Okimoto, Ryosuke Tanino, Yoshitsugu Tajima, Mamoru Harada

**Affiliations:** 1 Department of Digestive and General Surgery, Shimane University Faculty of Medicine, Shimane, Japan; 2 Department of Surgery, Hygo Prefectural Kakogawa Medical Center, Hyogo, Japan; 3 Department of Immunology, Shimane University Faculty of Medicine, Shimane, Japan; 4 Division of Medical Oncology & Respiratory Medicine, Department of Internal Medicine, Shimane University Faculty of Medicine, Shimane, Japan; University of South Alabama Mitchell Cancer Institute, UNITED STATES

## Abstract

Autophagy contributes to the treatment-resistance of many types of cancers, and chloroquine (CQ) inhibits autophagy. The tumor necrosis factor (TNF)-related apoptosis-inducing ligand (TRAIL) kills cancer cells but is minimally cytotoxic to normal cells. However, because the therapeutic efficacy of TRAIL is limited, it is necessary to augment TRAIL-induced anti-tumor effects. In this study, we explored the anti-tumor effects of a combination of CQ and TRAIL on two human pancreatic cancer cell lines: TRAIL-sensitive MiaPaCa-2 cells and Panc-1 cells that are less sensitive to TRAIL. Although both CQ and TRAIL reduced cancer cell viability in a dose-dependent manner, the combination acted synergistically. CQ increased the expression level of type-II LC3B without decreasing the expression of p62, an autophagic substrate, thus indicating inhibition of autophagy. CQ did not increase the levels of death receptors on cancer cells but reduced the expression of anti-apoptotic proteins. A combination of CQ and TRAIL significantly increased cancer cell apoptosis. CQ induced cell-cycle arrest in the G2/M phase. Also, CQ increased the p21 level but reduced that of cyclin B1. A combination of CQ and TRAIL reduced the colony-forming abilities of cancer cells to extents greater than either material alone. In xenograft models, combination CQ and TRAIL therapy significantly suppressed the growth of subcutaneously established MiaPaCa-2 and Panc-1 cells, compared with the untreated or monotherapy groups. Together, the results indicate that CQ in combination with TRAIL may be useful to treat human pancreatic cancer.

## Introduction

Autophagy has received a great deal of attention as a mechanism whereby cancer cells become resistant to therapy. Autophagy plays a fundamental role in protecting cells under conditions of starvation and stress [1]. However, these functions can render cancer cells therapy-resistant [2, 3]. We previously reported that autophagy inhibited apoptosis of human prostate and breast cancer cells treated with an innate adjuvant receptor ligand, poly (I:C) [4, 5]. In addition, many reports have suggested that inhibition of autophagy can restore susceptibility to anti-cancer therapies [6–8]. Several reports have also indicated that inhibition of autophagy increases the sensitivity of human cancer cells to the tumor necrosis factor (TNF)-related apoptosis-inducing ligand (TRAIL) [9–11]. In support of this notion, we previously reported that pifithrin-μ, which inhibits both HSP70 and autophagy, enhanced the TRAIL-induced antitumor effects on human pancreatic cancer cells [12]. In terms of clinical relevance, both chloroquine (CQ) and hydroxychloroquine (HCQ) may be useful drugs to inhibit autophagy. Both have been used to counter malaria and rheumatic arthritis [13, 14], and are known to be clinically safe. Moreover, HCQ has been used to treat several types of solid cancers in combination with other anti-cancer drugs [15, 16].

Apoptosis of cancer cells is induced primarily via two major pathways: the extrinsic and intrinsic pathways [17, 18]. TRAIL delivers death signals via the extrinsic apoptotic pathway, but also invokes the intrinsic mitochondrion-mediated pathway [18]. Therapeutically, TRAIL induces cancer cell death but is essentially non-toxic to normal cells [18]. TRAIL receptors are both positive and negative in nature: the death receptors (DRs) DR4 and DR5 engage in pro-apoptotic signaling, whereas the decoy receptors (DcRs) DcR1 and DcR2 competitively inhibit apoptotic signaling [18]. Normal cells are TRAIL-resistant because they preferentially express the DcRs [19]. Thus, the DRs were expected to be promising targets of anti-cancer therapy [20, 21]. However, cancer cells frequently exhibit TRAIL-resistance. Many resistance mechanisms have been reported [22], and efficient means of overcoming the problems are urgently required.

In the present study, we investigated the effects of CQ, an inhibitor of autophagy, on the TRAIL-sensitivity of two human pancreatic cancer cell lines: the TRAIL sensitive MiaPaCa-2 line and the Panc-1 line that is less sensitive to TRAIL. We found that CQ effectively sensitized these cancer cell lines to TRAIL. CQ promoted TRAIL-induced apoptosis, at least partially via downregulating anti-apoptotic proteins, and induced cell cycle arrest at the G2/M phase. Our findings suggest that inhibition of autophagy by CQ, in combination with TRAIL, may be a promising treatment for pancreatic cancer.

## Materials and methods

### Cell lines and reagents

Two human pancreatic cancer cell lines (MiaPaCa-2 and Panc-1) were kindly provided by Dr. K. Takenaga (Shimane University Faculty of Medicine) and were maintained in Dulbecco’s modified Eagle’s Medium (DMEM; Sigma-Aldrich, St. Louis, MO, USA) supplemented with 10% (v/v) fetal calf serum (InvitroGen, Grand Island, NY, USA) and 20 μg/mL gentamicin (Sigma-Aldrich). PrEC is a human normal prostate epithelial cell line purchased from Lonza (Walkersville, MD, USA). Human recombinant TRAIL was purchased from PeproTech, Rocky Hill, NJ, USA. CQ was purchased from InvivoGen.

### Cell viability assay

Cell viability was analyzed using the WST-8 assay (Nacalai Tesque, Kyoto, Japan). At the end of the recommended incubation period, 10 μL of WST-8 solution was added to each well, and the plates incubated for an additional 3 h. Absorbances at 560 nm were measured using a microplate reader (Beckman Coulter, Brea, CA, USA).

### Flow cytometric analysis

To explore DR4 (CD261) and DR5 (CD262) expression, cells were incubated with either an anti-DR4 or anti-D5 antibody (eBioscience, San Diego, CA, USA), followed by addition of FITC-conjugated goat anti-mouse IgG (H+L) (KPL, Gaithersburg, MD, USA). Mouse IgG served as a control. Cell death was assessed using an Annexin V-FITC Apoptosis Detection Kit (BioVision, Mountain View, CA, USA) with propidium iodide (PI) staining. A BrdU/7AAD Proliferation Kit (Becton Dickinson, Fullerton, CA, USA) was used according to the manufacturer’s instructions to evaluate cancer cell cycles and proliferation. All analyses were performed with the aid of a FACSCalibur flow cytometer (Becton Dickinson, Franklin Lakes, NJ, USA).

### Immunoblotting

Cells were lysed using a mammalian protein extraction reagent (M-PER; Thermo Scientific, Rockford, IL, USA) containing a protease inhibitor cocktail (Nacalai Tesque). Equal amounts of protein were resolved on 4–12% (w/v) gradient or 12% (w/v) SDS-PAGE gels and transferred to polyvinylidene fluoride membranes. The membranes were blocked and then incubated with the following primary antibodies: anti-LC3B (Cell Signaling Technology [CST], Danvers, MA, USA), anti-p62/SQSTM1 (CST), anti-Beclin-1 (CST), anti-Bcl-xL (BioLegend, San Diego, CA, USA), anti-caspase-3 (CST), anti-caspase-8 (Medical and Biological Laboratories, Nagoya, Japan), anti-caspase-9 (CST), anti-cyclin B1 (Santa Cruz Biotechnology, Santa Cruz, CA, USA), anti-cFLIP_S/L_ (Santa Cruz Biotechnology), anti-XIAP (CST), anti-p21 (CST), anti-β-actin (BioLegend), and α-tubulin (Santa Cruz Biotechnology). After washing, the membranes were incubated at room temperature for 30 min with either goat anti-rabbit or goat anti-mouse alkaline phosphatase-conjugated secondary antibody (Invitrogen). Protein bands were visualized using the CDP-star chemiluminescence technique and imaged with the aid of an ImageQuant LAS-4000 system (FujiFilm, Tokyo, Japan). The band intensities were scanned and quantified using ImageJ software (http://rsb.info.nih.gov/ij/).

### Colony-forming assay

Cells were seeded into flat-bottomed six-well plates and the indicated doses of TRAIL and CQ added. Two days later, the medium was replaced with new medium lacking TRAIL and CQ, and culture was continued for a further 8 days. Colonies were fixed in methanol, stained with 0.05% (w/v) crystal violet, and counted.

### *In vivo* xenograft model

BALB *nu/nu* female mice were purchased from CLEA Japan (Tokyo, Japan) and maintained under specific-pathogen-free conditions. The study protocol was approved by the Committee on the Ethics of Animal Experiments of the Shimane University Faculty of Medicine (permit nos.: IZ26-193, IZ29-19). All efforts were made to minimize suffering. Mice were inoculated subcutaneously (s.c.) in the right flank with 5×10^6^ MiaPaCa-2 cells or 2×10^6^ Panc-1 cells suspended in Matrigel (Japan BD Biosciences, Tokyo, Japan) at a 1:1 volume ratio in a volume of 100 μL. When the tumor diameter reached about 5 mm, the mice were injected intraperitoneally (i.p.) with CQ (50 mg/kg for MiaPaCa-2 and 100 mg/kg for Panc-1) daily for 6 days. TRAIL (1 μg for MiaPaCa-2 and 4 μg for Panc-1) in a volume of 50 μL was injected intratumorally (i.t.) on days 2 and 5 after initiation of CQ therapy. The vehicle control for i.t. injections was 50 μL of PBS. Subsequently, tumor size, 0.5 × length × width^2^, and body weight were measured twice weekly.

### Statistical analyses

Data were analyzed using the unpaired two-tailed Student’s *t-*test (for between-group comparisons) or via analysis of variance (ANOVA) with Tukey’s post hoc test (for among-group comparisons). A *P-*value <0.05 was considered to reflect statistical significance.

## Results

### CQ increases the TRAIL-sensitivity of two pancreatic cancer cell lines

**Fig 1:**
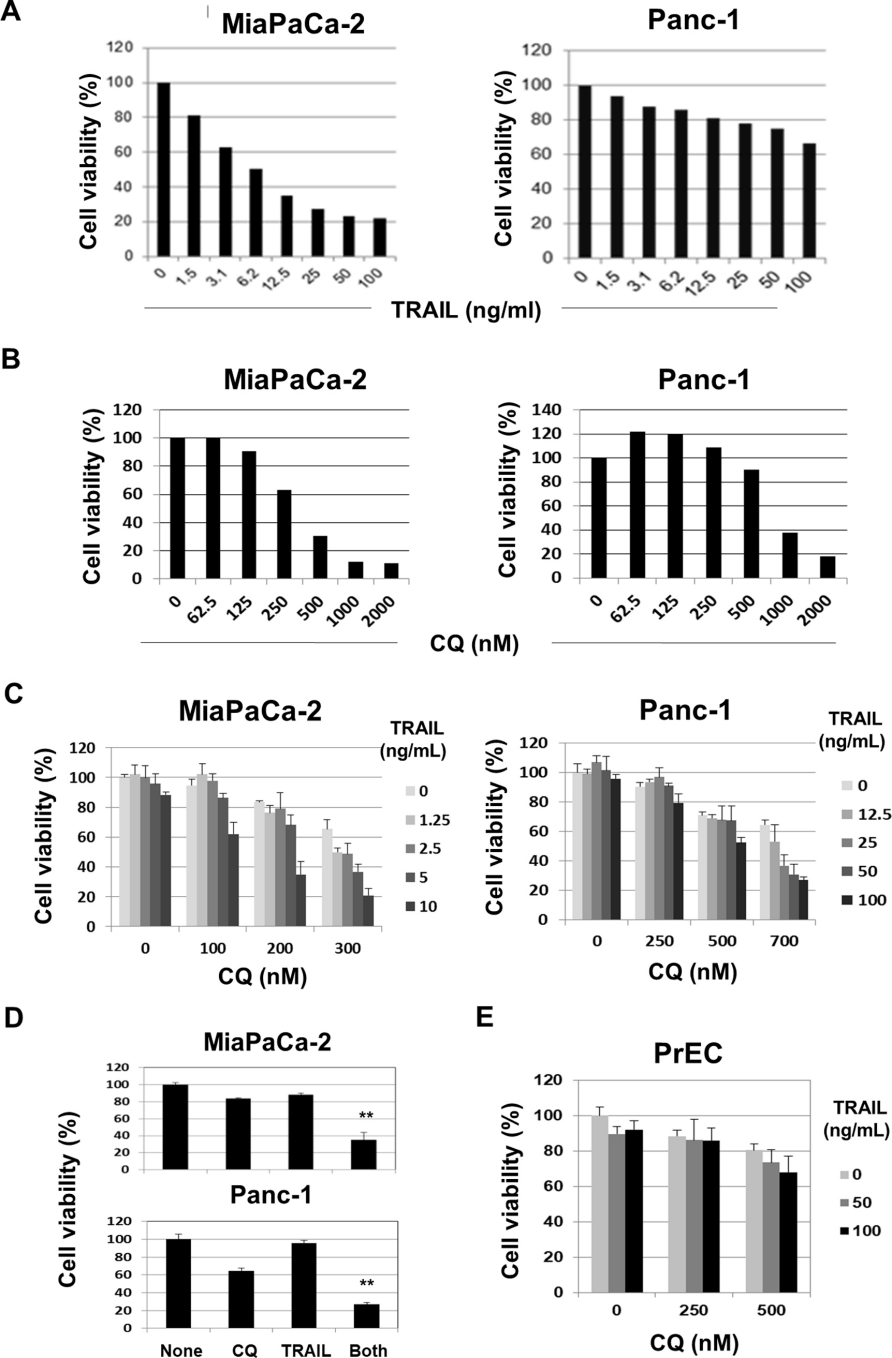
Effects of a combination of TRAIL and CQ on two human pancreatic cancer cell lines. (A) Two human pancreatic cancer lines were cultured in the presence of TRAIL. After 48 h, cell viabilities were determined by the WST-8 assay. The data represent the mean values of those of three wells. (B) Two cancer lines were cultured in the presence of CQ. After 48 h, cell viabilities were determined by the WST-8 assay. The data represent the mean values of those of three wells. (C) Two cancer cell lines were cultured with either or both of TRAIL and CQ at the indicated doses. After 48 h, cell viabilities were determined by the WST-8 assay. The data represent the mean values and standard deviation (SD) of those of three wells. (D) Representative results: CQ (200 nM) and TRAIL (10 ng/mL) for MiaPaCa-2 cells and CQ (700 nM) and TRAIL (100 ng/mL) for Panc-1 cells. The data represent the mean values and SD of those of three wells. **P<0.01. (E) Human normal prostate epithelial cells were cultured with either or both TRAIL and CQ at the indicated doses. After 48 h, cell viabilities were determined by the WST-8 assay. The data represent the mean values and SD of three wells.

We first examined the sensitivity of the two human pancreatic MiaPaCa-2 and Panc-1 cancer cell lines to TRAIL, which reduced the viability of MiaPaCa-2 cells in a dose-dependent manner. However, TRAIL exerted minimal effects on Panc-1 cells (Fig 1A). Thus, MiaPaCa-2 cells were highly sensitive but Panc-1 cells were less sensitive to TRAIL. CQ reduced the viability of both cell lines in a dose-dependent manner (Fig 1B). MiaPaCa-2 cells were more sensitive to CQ than were Panc-1 cells. We next examined the effects of TRAIL and CQ combinations (Fig 1C). CQ (200 or 300 nM) reduced the viability of MiaPaCa-2 cells when TRAIL was present at 10 ng/mL. Also, CQ (700 nM) reduced the viability of Panc-1 cells at doses of 50 and 100 ng/mL TRAIL. Representative results are shown in Fig 1D. The combination of CQ (200 nM) and TRAIL (10 ng/mL) significantly reduced the viability of MiaPaCa-2 cells compared with other combinations. Similarly, the combination of CQ (700 nM) and TRAIL (100 ng/mL) significantly reduced the viability of Panc-1 cells compared with other combinations. We also examined the effects of TRAIL and CQ combinations on a human normal prostate epithelial cell line, PrEC (Fig 1E). Although a higher dose of CQ decreased the viability of PrEC cells, CQ did not enhance the TRAIL-sensitivity of these normal cells. Thus, CQ effectively increased the TRAIL-sensitivity of both pancreatic cancer cell lines without definite effects on normal cells.

### CQ reduces the expressions of anti-apoptotic proteins in pancreatic cancer cell lines

**Fig 2:**
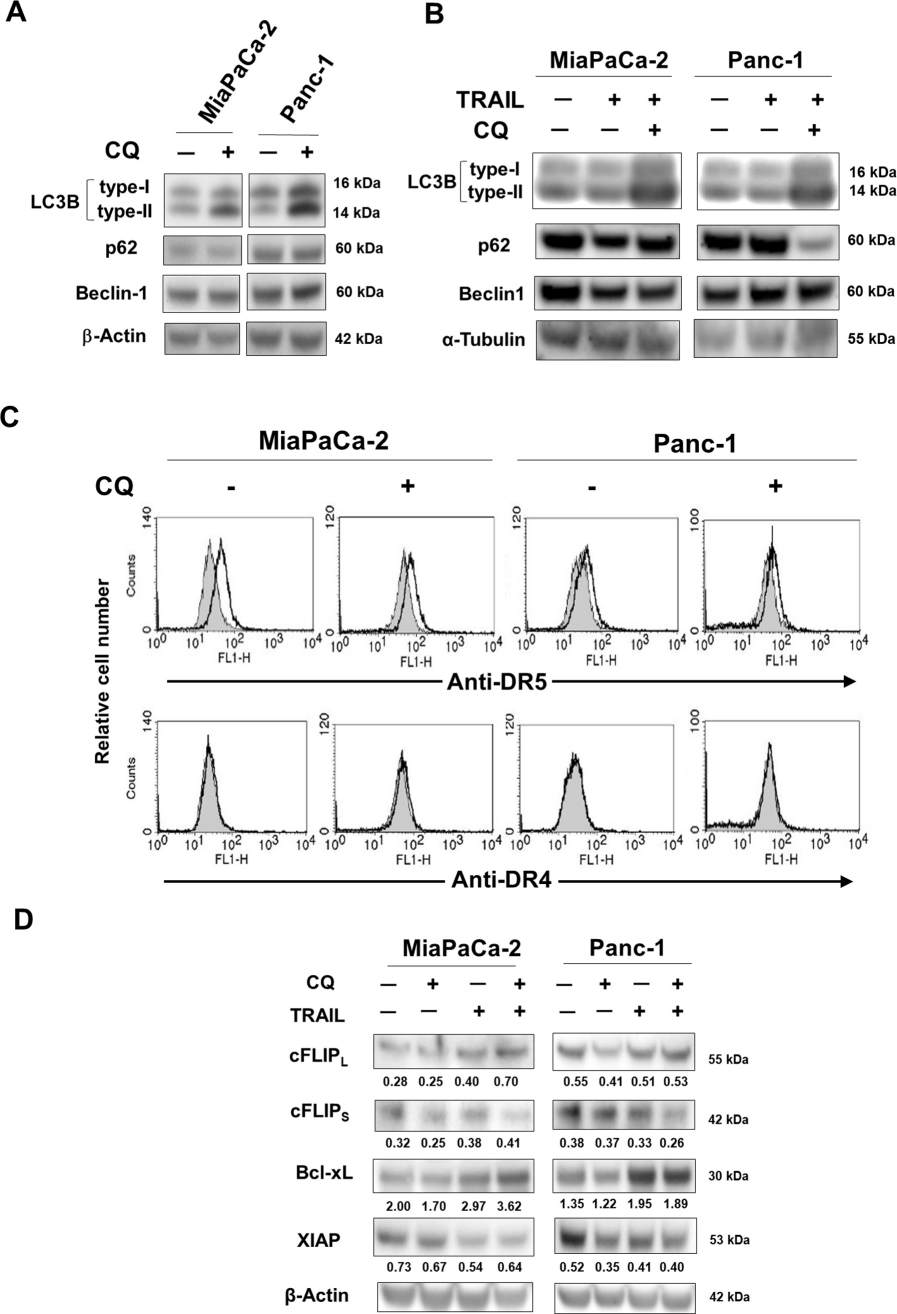
Effects of CQ on the levels of LC3B, anti-apoptotic proteins, and DRs, of two human pancreatic cancer cell lines. (A, B) Two cancer cell lines were cultured with CQ (200 nM for MiaPaCa-2 and 700 nM for Panc-1) and/or TRAIL (10 ng/mL for MiaPaCa-2 cells, 100 ng/mL for Panc-1 cells) for 24 h, lysed, and the expression levels of LC3B, p62, and Beclin-1 were measured. β-Actin and α-tubulin served as controls. (C) Two cancer lines were cultured in the presence of CQ (200 nM for MiaPaCa-2 and 700 nM for Panc-1). After 48 h, DR5 and DR4 expression levels were examined by flow cytometry. The lines exhibited binding of mAbs specific for either DR5 or DR4, followed by binding of an FITC-conjugated secondary antibody. The solid gray indicates binding of an isotype-matched control mouse IgG, followed by binding of an FITC-conjugated secondary antibody. (D) Two cancer cell lines were cultured with CQ (200 nM for MiaPaCa-2 and 700 nM for Panc-1 cells) for 48 h, and the expression levels of cFLIP, Bcl-xL, and XIAP were measured. β-Actin served as a control. The number represents the band density of each protein, which was normalized to the β-actin loading control. Similar results were obtained in two independent experiments.

CQ is known to inhibit autophagy via inhibition of lysosomal function, and we confirmed this effect. As shown in Fig 2A, CQ increased the expression of type-II LC3B [23] in both cell lines, whereas no clear change was observed in terms of p62 expression; this protein is a substrate of autophagy-mediated degradation [24]. Similarly, no change was observed in terms of Beclin-1 expression. In addition, we examined the expression of these molecules when treated with TRAIL alone or TRAIL and CQ (Fig 2B). The combination of TRAIL and CQ promoted autophagy in both cell lines, and the expression of p62 in Panc-1 cells was significantly decreased. In this regard, we have previously reported that siRNA-mediated knockdown of Beclin-1 in these cell lines increased sensitivity to TRAIL [12]. Alternatively, CQ did not notably affect the expression of either DR4 or DR5 in either cell line (Fig 2C). cFLIP inhibits the activation of caspase-8 [25, 26], which is a key caspase of TRAIL-induced apoptosis. We also recently reported that the Bcl-xL expression level was negatively correlated with the TRAIL-sensitivity of a panel of human pancreatic cancer cell lines [27]. In addition, XIAP has been reported to inhibit TRAIL-induced apoptosis [28]. Therefore, we examined the effects of CQ on the expression of these proteins (Fig 2D). CQ reduced the expression of cFLIP_S_ in MiaPaCa-2 and that of cFLIP_L_ in Panc-1 cells. CQ also reduced the expression of Bcl-xL in both cell lines and that of XIAP in Panc-1 cells. However, the combination of CQ and TRAIL increased the expression of cFLIP_L_ in MaiPaCa-2 cells and that of Bcl-xL in both cell lines. This opposite result suggests the possibility that TRAIL-induced cell death occurs preferentially in cells that reduced anti-apoptotic proteins after treatment with CQ. Nevertheless, CQ was suggested to reduce the expression of anti-apoptotic proteins in these pancreatic cancer cell lines.

### CQ augments TRAIL-induced apoptosis and induces G2/M phase arrest of cancer cells

**Fig 3:**
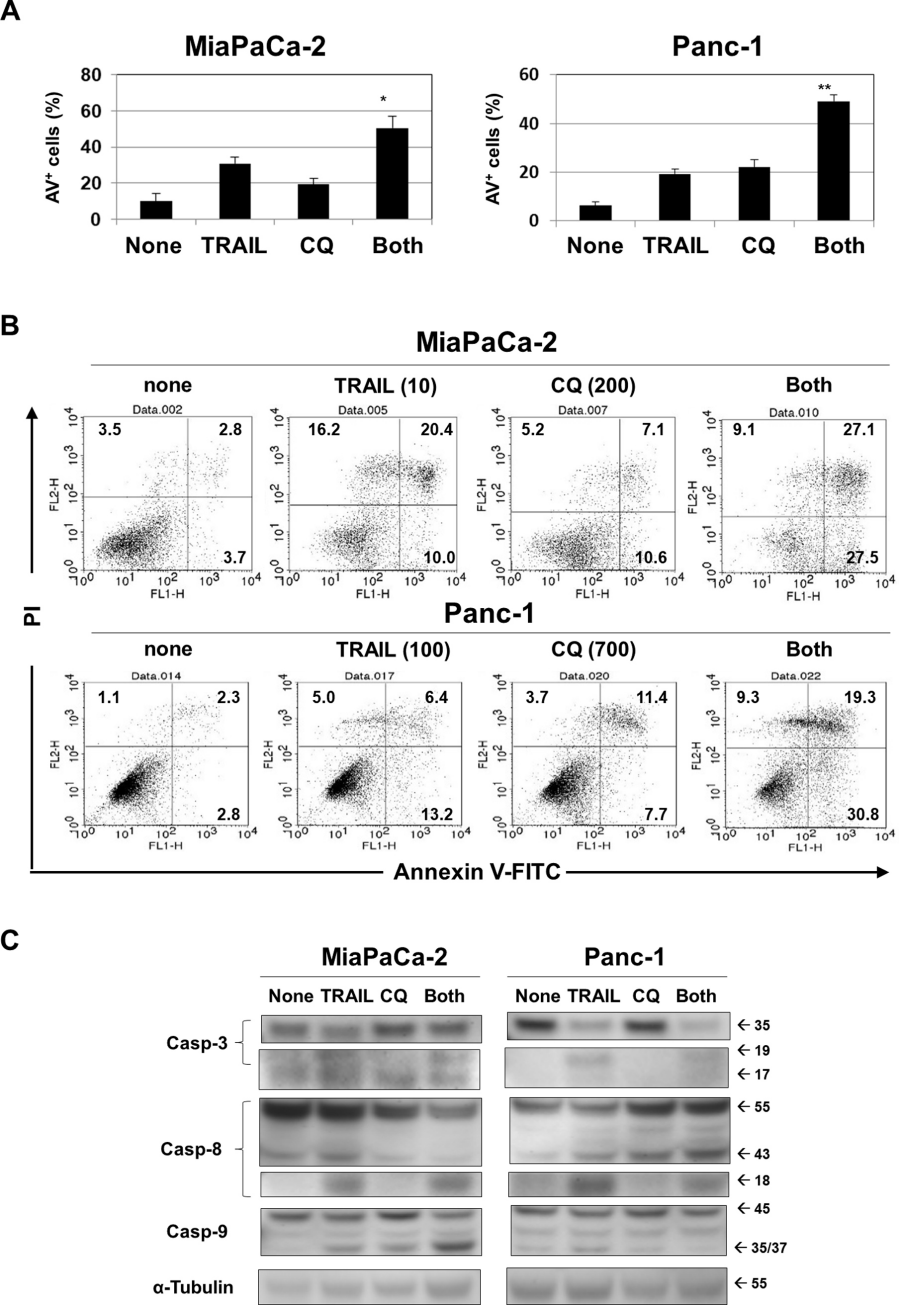
Apoptosis of cancer cells treated with TRAIL and CQ. (A) Two cancer cell lines were cultured with the indicated doses of TRAIL and CQ for 48 h. After staining with Annexin V-FITC/PI, the cells were subjected to flow cytometry. The data are the means and standard deviation (SD) of those from three wells. *P<0.05. **P<0.01. (B) Representative results. The numbers indicate the proportions of cells in each subset. (C) Two cancer cell lines were cultured with CQ (200 nM for MiaPaCa-2 cells, 700 nM for Panc-1 cells), TRAIL (10 ng/mL for MiaPaCa-2 cells, 100 ng/mL for Panc-1 cells), or both for 24 h. The expression levels of caspase-3, caspase-8, and caspase-9 were measured. α-Tubulin served as a control.

Cell death and growth arrest reduce cell viability when analyzed by the WST-8 assay. We next sought to understand the mechanisms of the effects induced by CQ and TRAIL. As shown in Fig 3A, although TRAIL or CQ alone increased the proportions of apoptotic cells, drug combinations further increased these proportions in both cancer cell lines. Representative results are shown in Fig 3B. In addition, the combination of TRAIL and CQ increased the expression of cleaved caspase-9 in MiaPaCa-2 and cleaved caspase-8 in Panc-1, respectively (Fig 3C), These results indicate that a combination of these drugs can stimulate intrinsic and extrinsic apoptosis pathways of MiaPaCa-2 and Panc-1, respectively.

**Fig 4:**
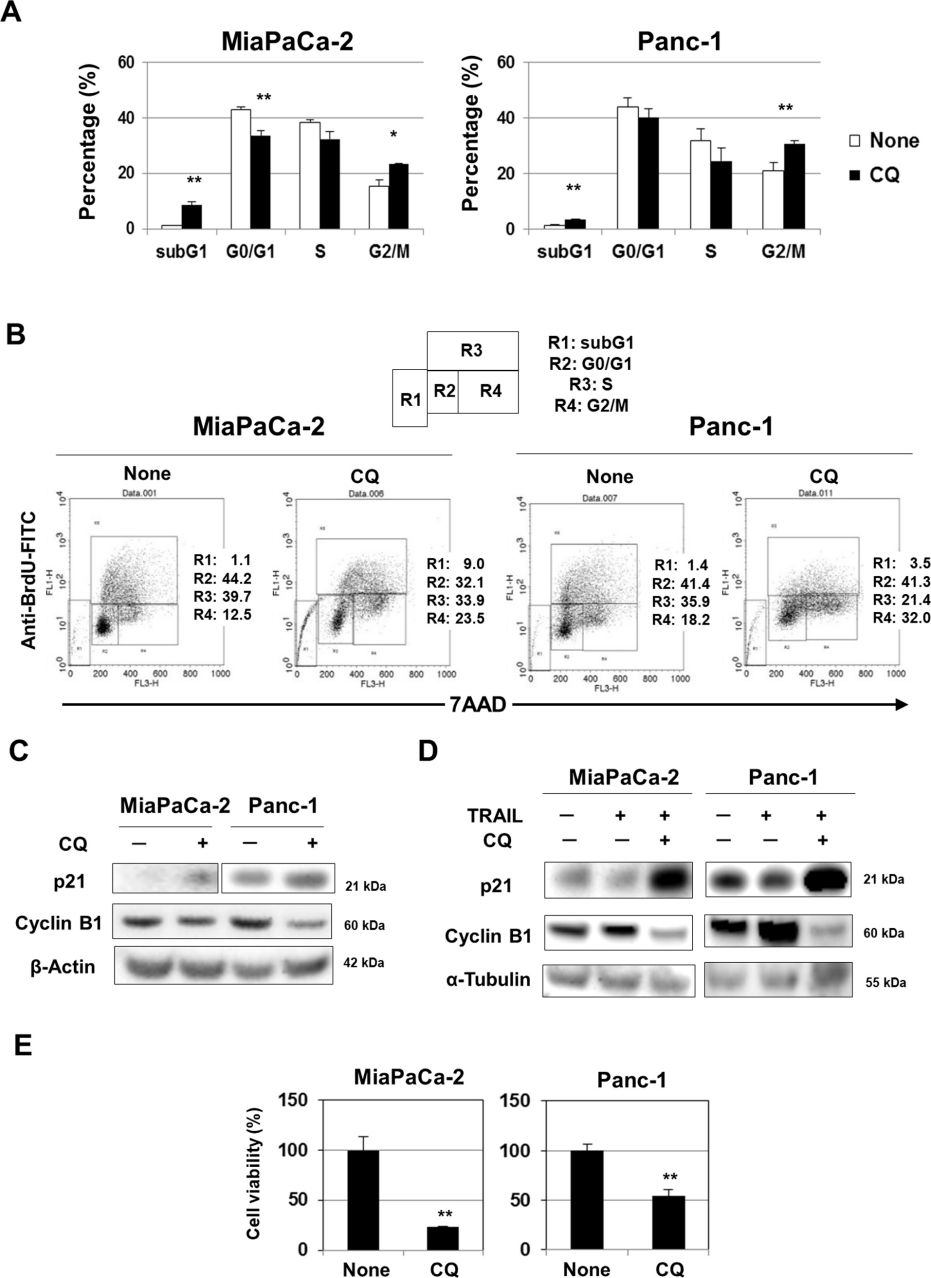
G2/M arrest of CQ-treated pancreatic cancer cell lines. (A) Two cell lines were cultured with CQ (200 nM for MiaPaCa-2 and 700 nM for Panc-1 cells) for 2 days. After staining with anti-BrdU-FITC/7AAD, the cells were subjected to flow cytometry. We present a summary of the results. All data points are the means and standard deviation (SD) of data from three wells. *P<0.05, **P<0.01. (B) Representative results. (C, D) Two cancer cell lines were cultured with CQ (200 nM for MiaPaCa-2 and 700 nM for Panc-1 cells) and/or TRAIL (10 ng/mL for MiaPaCa-2 cells, 100 ng/mL for Panc-1 cells) for 48 h and the expression levels of p21 and cyclin B1 were measured. β-Actin and α-tubulin served as controls. (E) Two cancer cell lines were cultured with CQ (200 nM for MiaPaCa-2 and 700 nM for Panc-1 cells) for 2 days, and then for a further 6 days in the absence of CQ. Cell viability was determined by the WST-8 assay. The data shown represent the means and SD of those from three wells. **P<0.01.

We also performed cell cycle assays (Fig 4A). In both cell lines, CQ alone significantly increased sub-G1 cell subsets, implying that apoptosis was in play. When added to MiaPaCa-2 cells, CQ decreased the proportions in the G0/G1 phase and increased those in the G2/M phase. Similarly, CQ increased the proportions of Panc-1 cells in the G2/M phase. Representative results are shown in Fig 4B, and suggest that CQ induced cell cycle arrest at the G2/M phase. In line with this suggestion, CQ increased p21 expression and reduced cyclin B1 expression (Fig 4C). These changes were enhanced in the presence of TRAIL (Fig 4D). Additionally, short-term (2-day) treatment with CQ reduced the viability of cancer cells over an additional 6 days of culture in the absence of CQ (Fig 4E), indicating that CQ not only induced apoptosis to a certain extent, but also cell cycle arrest at G2/M phase in both cell lines.

### Combinations of CQ and TRAIL inhibit the colony-forming capacities of pancreatic cancer cell lines

**Fig 5:**
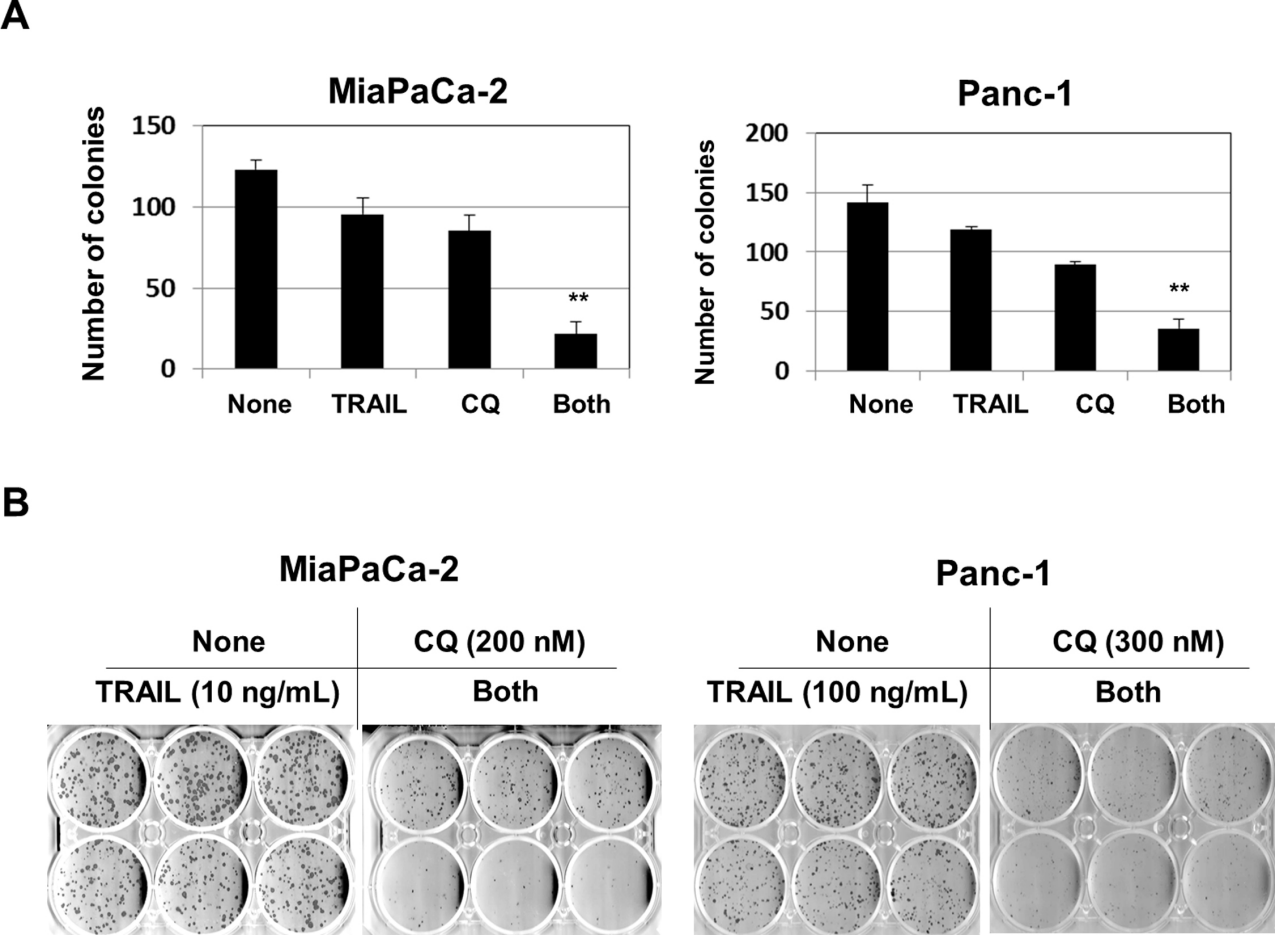
Effects of TRAIL and CQ combinations on the colony-forming abilities of cancer cells. (A) MiaPaCa-2 and Panc-1 cells were cultured with the indicated doses of TRAIL and/or CQ. After 48 h, the supernatants were replaced with drug-free medium and growth continued for 8 days. Colonies were stained with crystal violet and counted. Each group featured three wells. **P<0.01. (B) Photograph of plates.

We next examined the effects of CQ and TRAIL combinations on the colony-forming capacities of the two cancer cell lines. Both lines were initially cultured with either or both of CQ and TRAIL for 2 days, and subsequently cultured without either drug. When Panc-1 cells were cultured for 2 days with CQ at 700 nM, colony formation was completely inhibited; we thus reduced the dose to 300 nM. Either TRAIL or CQ reduced colony numbers slightly, whereas drug combinations reduced colony numbers significantly, in both cell lines (Fig 5A). As shown in Fig 5B, the colony sizes of CQ-treated cells were smaller than those of untreated or TRAIL-treated cells, suggesting that CQ arrested the growth of cancer cells.

### The effects of TRAIL and CQ combination therapy on the *in vivo* growth of MiaPaCa-2 and Panc-1 cells

**Fig 6:**
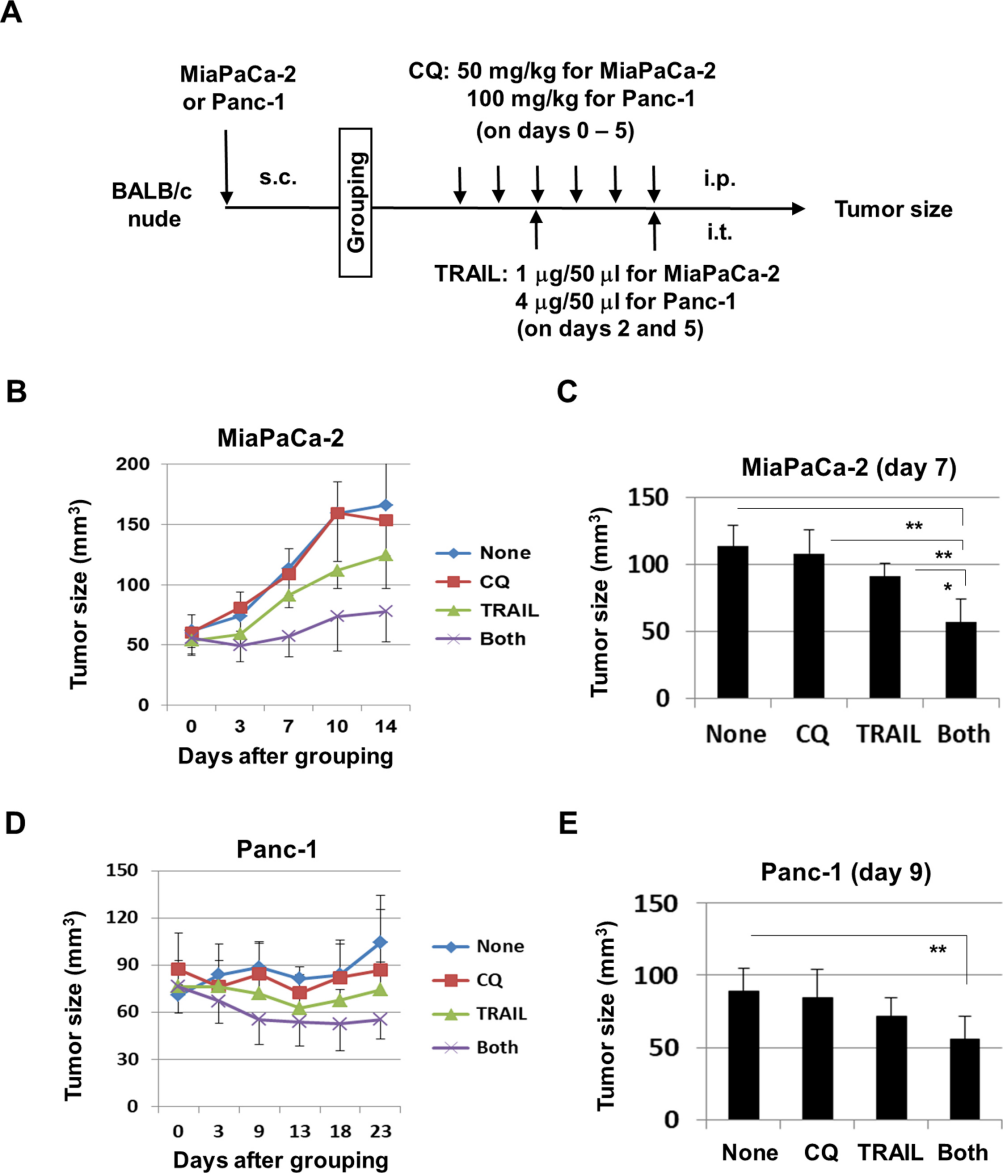
*In vivo* anti-tumor effects of CQ and TRAIL on the growth of MiaPaCa-2 and Panc-1 cells. (**A**) Treatment protocol. (B) BALB *nu/nu* female mice were inoculated in the right flanks with 5 × 10^6^ MiaPaCa-2 cells suspended in Matrigel. When the tumor diameters reached approximately 5 mm, the mice were pooled and divided into four groups. On days 0, 1, 2, 3, 4, and 5 thereafter, the mice received i.p. injections of CQ (50 mg/kg). The vehicle control was 200 μL PBS. On days 2 and 5 after grouping, TRAIL (1 μg/50 μL) was injected i.t.. The vehicle control was 50 μL PBS. Tumor sizes were measured on the indicated days. The data shown represent the means and standard deviation (SD) of 5 mice. (C) Tumor sizes of MiaPaCa-2 on day 7. *P<0.05, **P<0.01 (ANOVA). (D) Similarly, BALB *nu/nu* female mice were inoculated in the right flanks with 2 × 10^6^ Panc-1 cells, and treated with CQ (100 mg/kg) and/or TRAIL (4 μg/50 μL). The data shown represent the means and SD of six mice. (E) Tumor sizes of Panc-1 on day 9. **P<0.01 (ANOVA).

Finally, we examined the *in vivo* effects of CQ and TRAIL combinations using mice hosting MiaPaCa-2 or Panc-1 xenografts. The treatment protocol is shown in Fig 6A. In terms of MiaPaCa-2, the tumor size in mice given combination therapy was smaller than that in untreated mice or those given monotherapies (Fig 6B). At 7 days after therapy initiation, the tumor size in mice given combination therapy was significantly smaller than those in mice given the monotherapies (Fig 6C). However, the body weights did not differ significantly (S1A Fig). Panc-1-bearing mice were treated with higher doses of CQ and TRAIL because Panc-1 cells were more resistant to these drugs *in vitro* compared with MiaPaCa-2 cells (Fig 1A and 1B). Combination therapy significantly inhibited the tumor growth of Panc-1 cells in comparison with the untreated group (Fig 6D and 6E). Representative photographs are shown in S1B Fig. The CQ treatment with or without TRAIL transiently decreased the body weight of Panc-1-bearing mice, which was soon recovered (S1C Fig). We examined the expression of cleaved caspase-3 and LC3B in the tumor tissues from the MiaPaCa-2-bearing mice that were treated with the protocol of Fig 6A. However, no definite change was observed among the groups (S1D Fig). Taking the findings together, a combination of TRAIL and CQ could suppress the growth of two human pancreatic cancer cell lines *in vivo*.

## Discussion

Autophagy plays multiple roles in cell biology. The fundamental role is cytoprotection [9], which exerts negative effects on anti-cancer therapy. Many reports have shown that inhibition of autophagy enhances anti-tumor therapeutic efficacies [10, 11, 29–32]. This is true of pancreatic cancer cells; autophagy increased the resistance to therapy of pancreatic cancer cells [12, 33, 34]. To this end, we utilized CQ to inhibit autophagy; CQ has been clinically used as an anti-malarial and an anti-rheumatic [13, 14]. CQ is a lysosomotropic agent preventing the endosomal acidification that activates lysosomal enzymes requiring an acidic pH, and preventing the fusion of endosomes and lysosomes. In the present study, we confirmed that CQ inhibited autophagy. CQ increased the level of type-II LC3B protein but had no effect on that of p62, an autophagic substrate [24], in either pancreatic cancer cell line (Fig 2A).

How does CQ enhance TRAIL-induced apoptosis of pancreatic cancer cells? The DRs and DcRs are agonistic and antagonistic TRAIL receptors, respectively [17]. CQ did not increase the cell surface expression levels of the DRs (Fig 2B), and we previously reported that both cell lines were negative for the DcRs [27]. TRAIL-sensitivity of human pancreatic cancer cell lines is negatively correlated with the extent of Bcl-xL expression [27]. In this study, CQ decreased the expression of Bcl-xL, while CQ and TRAIL combination increased its expression in both cell lines (Fig 2C). These unexpected results could be explained by the idea that TRAIL-induced apoptosis preferentially occurs in cells with decreased Bcl-xL expression after CQ treatment. In addition, several reports have indicated that XIAP of cancer cells imparts TRAIL-resistance [28], and CQ reduced XIAP expression in both cell lines (Fig 2C). cFLIP_L/S_ are inhibitors of caspase-8 [25, 26], and CQ treatment also decreased cFLIP_S_ expression in MiaPaCa-2 cells and the cFLIP_L_ expression in Panc-1 cells, respectively (Fig 2C). These results indicate that such reductions of anti-apoptotic proteins may explain, at least in part, why CQ promoted TRAIL-induced apoptosis. However, our results contradict those of other reports indicating that activation of drug-mediated autophagy promotes the degradation of cFLIP and other anti-apoptotic proteins, triggering apoptosis and/or increasing TRAIL-sensitivity [35–37]. The cited studies used human non-small-cell lung cancer cells, whereas we utilized human pancreatic cancer cell lines. However, we do not yet have a clear explanation of the contradictory results.

Caspase-8 is a key enzyme of the extrinsic apoptotic pathway, activation of which triggers Bid cleavage. Truncated Bid activates the mitochondrion-mediated pathway of intrinsic apoptosis [38]. Intriguingly, autophagy features caspase-8 degradation [39]. Thus, if CQ prevented the autophagic degradation of caspase-8, TRAIL-induced apoptosis should be augmented. However, TRAIL-mediated apoptosis is regulated by various mechanisms [40]. In this study, we investigated whether combination therapy could stimulate the extrinsic or intrinsic apoptosis pathway to promote cancer cell death (Fig 3C). Interestingly, combination therapy induced activation of caspase-9 in MiaPaCa-2 cells while it induced activation of caspase-8 in Panc-1 cells. Accordingly, combination therapy with TRAIL and CQ stimulates the intrinsic apoptosis pathway in MiaPaCa-2 cells, while it stimulates the extrinsic apoptosis pathway in Panc-1 cells.

We also examined the cell cycles of CQ-treated cancer cells. CQ induced not only accumulation of apoptotic subG1-phase cells but also G2/M phase arrest (Fig 4A and 4B). Because p21 and cyclin B1 play positive and negative roles, respectively, in G2/M arrest [41,42], we examined the expression of these proteins in CQ-treated cancer cells and found that CQ increased the p21 level but decreased that of cyclin B1 (Fig 4C), compatible with G2/M phase arrest. To the best of our knowledge, this is the first report to show that CQ induces G2/M phase arrest in human pancreatic cancer cells. In addition, when cells were cultured with CQ for 2 days, subsequent proliferation in the absence of CQ was greatly reduced (Fig 4D). Short-term CQ treatment thus seemed to induce subsequent growth arrest of cancer cells. This may explain why CQ inhibited colony formation. The colony sizes of CQ-treated cancer cells were smaller than those of control cells (Fig 5B). Presumably, the CQ-induced growth arrest of cancer cells was in play even in the presence of TRAIL. Interestingly, CQ was reported to inhibit pancreatic cancer stem cells [43, 44]. Therefore, CQ may selectively suppress cancer stem cell growth in the colony-forming assay. Given that the presence of cancer stem cells is a major problem in many types of cancer [45], CQ may usefully reduce the numbers of such cells, potentiating other anti-cancer therapies. We plan to investigate the effects of combinations of CQ and TRAIL on pancreatic cancer stem cells.

We examined the effect of TRAIL and CQ combination therapy in xenograft models. The combination significantly reduced the growth of MiaPaCa-2 tumors, compared with either monotherapy (Fig 6B and 6C), but body weight (an indicator of general condition) was not affected (S1A Fig). In addition, although the *in vivo* growth of Panc-1 was slow and the combination therapy efficacy was low, the tumor growth was significantly suppressed by CQ and TRAIL combinations compared with the untreated group (Fig 6D and 6E and S1B Fig). We also examined the expression of cleaved caspase-3 and LC3B in the tumor tissues from the treated MiaPaCa-2-bearing mice (S1D Fig). However, no definite change was observed among the groups. Although we have no clear explanation on this result, apoptotic cells could have been rapidly processed by phagocytes *in vivo*.

In conclusion, we showed that the autophagic inhibitor CQ effectively sensitized human pancreatic cancer cells to TRAIL both *in vitro* and *in vivo*. In terms of the underlying mechanism, CQ promoted TRAIL-induced apoptosis and induced cell cycle arrest in the G2/M phase. These findings suggest that CQ may usefully augment the therapeutic efficacy of TRAIL against human pancreatic cancer.

## Supporting information

S1 Fig Effects of CQ and TRAIL combination on body weight and tumor growth in xenograft mouse models. (A) Body weights of nude mice bearing MiaPaCa-2 cells were measured on the indicated days. The data shown represent the means and standard deviation (SD) of five mice. (B) Representative photographs of Panc-1-bearing mice 23 days after grouping. The red arrow heads represent the tumor sites. (C) Body weights of nude mice bearing Panc-1 cells were measured on the indicated days. The data shown represent the means and SD of six mice. (D) On the next day after the last injection of CQ and/or TRAIL, MiaPaCa-2 tissues were harvested and examined for the expression of caspase-3 and LC3B. Each group consists of two mice. α-Tubulin served as a control.
(PDF)
