# Supplementary material for: Chloroquine augments TRAIL-induced apoptosis and induces G2/M phase arrest in human pancreatic cancer cells
Source: PLoS One. 2018 Mar 7;13(3):e0193990. doi: 10.1371/journal.pone.0193990 (PMC5841811; doi:10.1371/journal.pone.0193990)
Supplement: S1 Fig — (A) Body weights of nude mice bearing MiaPaCa-2 cells were measured on the indicated days. The data shown represent the means and standard deviation (SD) of five mice. (B) Representative photographs of Panc-1-bearing mice 23 days after grouping. The red arrow heads represent the tumor sites. (C) Body weights of nude mice bearing Panc-1 cells were measured on the indicated days. The data shown represent the means and SD of six mice. (D) On the next day after the last injection of CQ and/or TRAIL, MiaPaCa-2 tissues were harvested and examined for the expression of caspase-3 and LC3B. Each group consists of two mice. α-Tubulin served as a control. (PDF) [file pone.0193990.s001.pdf]

Supporting Information Figure S1

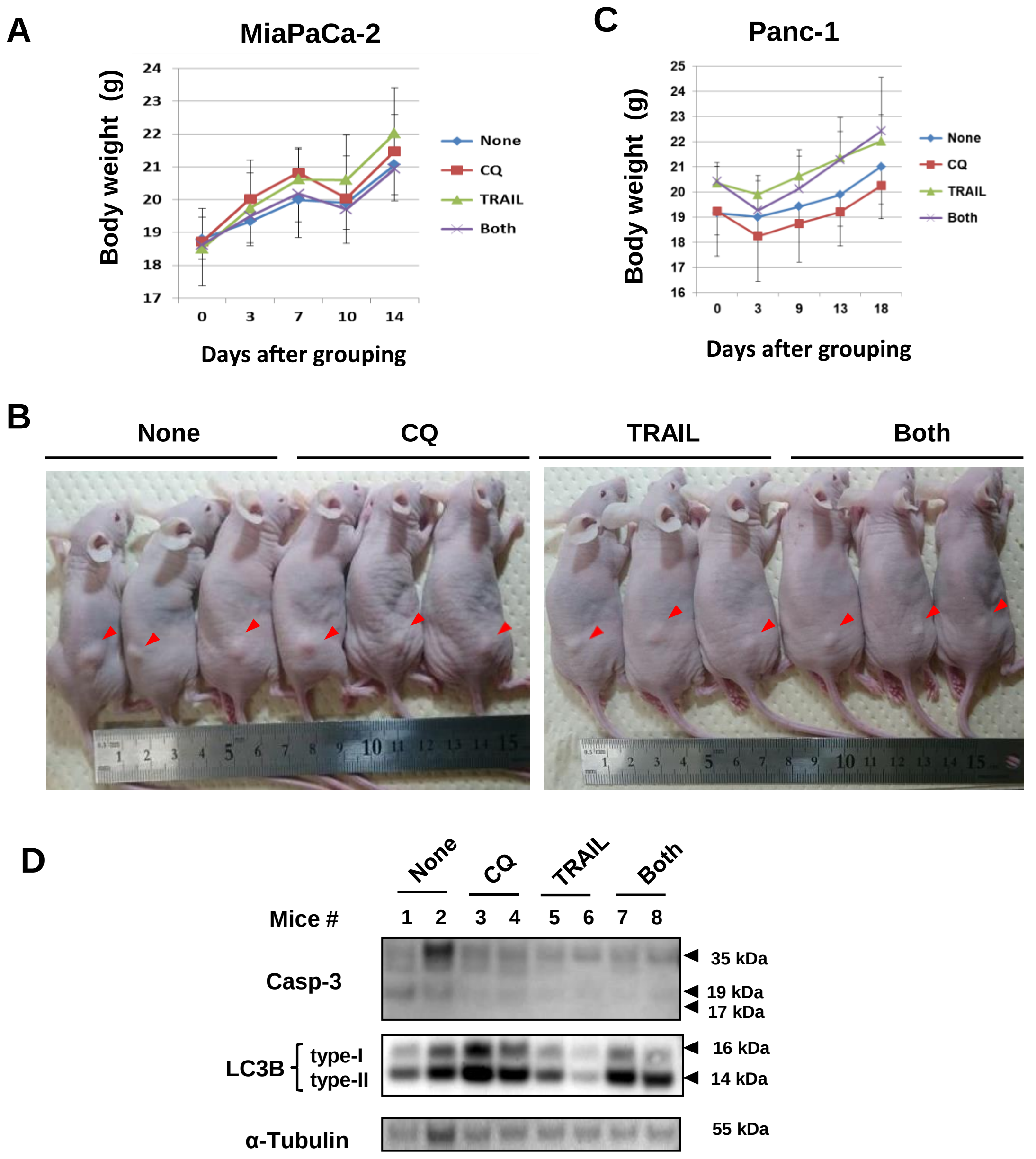

Effects of CQ and TRAIL combinations on body weight and tumor growth in xenograft mouse models

(A) Body weights of nude mice bearing MiaPaCa-2 cells were measured on the indicated days. The data shown represent the means and standard deviation (SD) of five mice. (B**)** Representative photographs of Panc-1-bearing mice 23 days after grouping. The red arrow heads represent the tumor sites. (C) Body weights of nude mice bearing Panc-1 cells were measured on the indicated days. The data shown represent the means and SD) of six mice. (D) On the next day after the last injection of CQ and/or TRAIL, MiaPaCa-2 tissues were harvested and examined for the expression of caspase-3 and LC3B. Each group consists of two mice. α-Tubulin served as a control.
